# Hypoxia and Hypoxia-Inducible Factors in Kidney Injury and Repair

**DOI:** 10.3390/cells8030207

**Published:** 2019-02-28

**Authors:** Shaoqun Shu, Ying Wang, Meiling Zheng, Zhiwen Liu, Juan Cai, Chengyuan Tang, Zheng Dong

**Affiliations:** 1 Department of Nephrology, The Second Xiangya Hospital of Central South University, Hunan Key Laboratory of Kidney Disease and Blood Purification, Changsha 410011, China; 168211042@csu.edu.cn (S.S.); 168211036@csu.edu.cn (Y.W.); zhengmeiling@sklmg.edu.cn (M.Z.); fry46152@163.com (Z.L.); cjane218@csu.edu.cn (J.C.); tangchengyuan@csu.edu.cn (C.T.); 2 The State Key Laboratory of Medical Genetics, School of Life Sciences, Central South University, Changsha 410011, China; 3 Department of Cellular Biology and Anatomy, Medical College of Georgia at Augusta University and Charlie Norwood VA Medical Center, Augusta, GA 30912, USA

**Keywords:** hypoxia, HIF, kidney injury, kidney repair, prolyl hydroxylase domain-containing protein (PHD)

## Abstract

Acute kidney injury (AKI) is a major kidney disease characterized by an abrupt loss of renal function. Accumulating evidence indicates that incomplete or maladaptive repair after AKI can result in kidney fibrosis and the development and progression of chronic kidney disease (CKD). Hypoxia, a condition of insufficient supply of oxygen to cells and tissues, occurs in both acute and chronic kidney diseases under a variety of clinical and experimental conditions. Hypoxia-inducible factors (HIFs) are the “master” transcription factors responsible for gene expression in hypoxia. Recent researches demonstrate that HIFs play an important role in kidney injury and repair by regulating HIF target genes, including microRNAs. However, there are controversies regarding the pathological roles of HIFs in kidney injury and repair. In this review, we describe the regulation, expression, and functions of HIFs, and their target genes and related functions. We also discuss the involvement of HIFs in AKI and kidney repair, presenting HIFs as effective therapeutic targets.

## 1. Introduction

Acute kidney injury (AKI), a major kidney disease with high morbidity and mortality, is characterized by a rapid loss of renal function [1]. The most common causes of AKI include sepsis, renal ischemia reperfusion (IR), and nephrotoxins. Pathologically, AKI is featured by sublethal and lethal injury of renal tubular epithelial cells [2,3]. Besides its acute effect on mortality, AKI can also contribute to the development and progression of chronic kidney disease (CKD) [4,5]. After initial injury, surviving renal proximal tubular epithelial cells (RPTCs) undergo dedifferentiation and proliferation to restore the integrity of renal tubules. However, when the injury becomes severe or episodic, the incomplete or maladaptive repair promotes the progression to CKD [6,7].

Hypoxia is a condition in which a cell or an organism has insufficient supply of oxygen. It occurs in microcirculation injury and hypoperfusion in tissues and organs including kidneys [8,9,10]. Hypoxia occurs in both AKI (not only the acute phase but also the recovery phase) and CKD under a variety of clinical and experimental conditions [11,12,13,14,15]. Meanwhile, it is also related to CKD-associated pathological states such as anemia [16] and inflammation [17]. The cellular response to hypoxia is centered on hypoxia-inducible factor (HIF). Under hypoxic condition, cells upregulate HIF, a well-known heterodimeric transcription factor that controls the transcription of more than 100 genes including erythropoietin (*EPO*), vascular endothelial growth factor (*VEGF*), and glucose transporter-1 (*GLUT1*) to restore tissue homeostasis by stimulating erythropoiesis, angiogenesis, anaerobic glycolysis, and other adaptive processes [18,19]. In kidneys, oxygen diffusion shunt between venous and arterial vessels that are in close physical relationship leads to a relatively low oxygen tension in kidney tissues, especially in renal medulla [20]. However, renal tubules are high in oxygen consumption [14]. Consequently, the low oxygen supply and high oxygen demand make the kidney vulnerable to hypoxia. Emerging evidence indicates that HIF and hypoxia response play an important role in various types of AKI [21,22,23] and CKD [14,15,24].

In this review, we will mainly concentrate on the function and mechanisms of HIF (HIF-1 and HIF-2) in kidney injury and repair, as they are the master transcriptional regulators responsive to hypoxia. We also discuss the therapeutic potential of targeting HIF for ameliorating kidney injury and accelerating kidney repair.

## 2. Regulation of HIF

**Figure 1:**
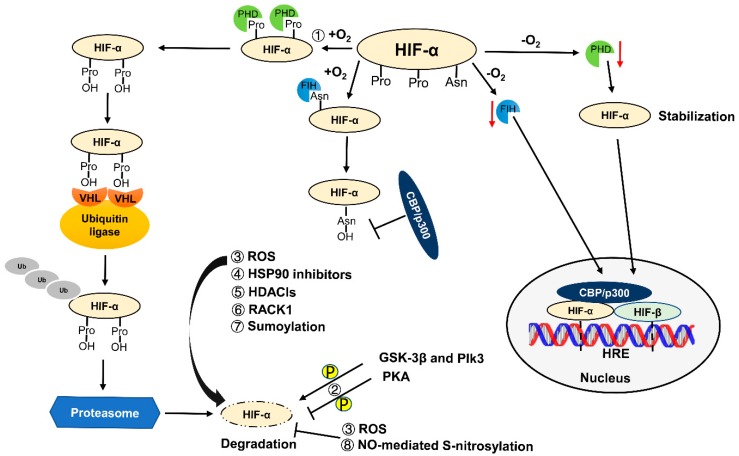
Regulation of the stability and transcription activity of HIF. In the presence of oxygen or normoxia, PHDs hydroxylate two prolyl residues of HIF-α. The hydroxylated HIF-α then binds to VHL-E3-ubiquitin ligase complex, leading to poly-ubiquitination and proteosomal degradation. Meanwhile, FIH hydroxylates an asparaginyl residue of HIF-α. Asparaginyl hydroxylated HIF-α prevents the recruitment of CBP/p300 coactivators, which is required for the full transcriptional activity of HIF. In the absence of O_2_ or hypoxia, PHD-mediated prolyl residue hydroxylation is inhibited, resulting in HIF-α stabilization. The stabilized HIF-α translocates into nucleus and then dimerizes with HIF-β to transactivate target genes. Meanwhile, FIH-mediated asparaginyl residue hydroxylation is also inhibited, causing the recruitment of CBP/p300 coactivators to enhance the transcription activity of HIF. In addition to oxygen, phosphorylation and ROS may play dual roles in HIF-α regulation; HSP90 inhibitors, HDACIs, RACK1, and sumoylation can decrease the stability of HIF-α, while NO-mediated S-nitrosylation can enhance the stability of HIF-α. Abbreviations: hypoxia-inducible factor (HIF), prolyl hydroxylase domain-containing protein (PHD), von Hippel–Lindau (VHL), factor inhibiting HIF (FIH), CREB-binding protein (CBP), hypoxia response element (HRE), reactive oxygen species (ROS), histone deacetylase inhibitors (HDACIs), receptor of activated protein kinase C 1 (RACK1), phosphorylation (P), glycogen synthase kinase-3beta (GSK-3β), polo-like kinase 3 (Plk3), protein kinase A (PKA), and nitric oxide (NO).

HIF is a protein heterodimer that is composed of an inducible α subunit (HIF-1α, HIF-2α, or HIF-3α) and a constitutively expressed subunit HIF-β. Therefore, the expression and function of HIF mainly depends on HIF-α. Notably, the stability of HIF-α is oxygen dependent. Under hypoxia, two critical prolyl residues of HIF-α are hydroxylated by specific prolyl hydroxylase domain-containing proteins (PHDs). Following prolyl hydroxylation, HIF-α binds to the von Hippel–Lindau protein (pVHL)-E3-ubiquitin ligase complex for ubiquitination and rapid degradation by proteasome [25,26] (Figure 1). All PHDs (PHD1, PHD2, and PHD3) are expressed in renal tubular epithelial cells [27]. Higher levels of PHDs are found in the thick ascending limb, distal convoluted tubule, and collecting duct of inner medulla, where oxygen tension is known to be physiologically low. PHD1 is expressed in the nucleus, while PHD2 is expressed in the cytoplasm, and PHD3 is found both in the nucleus and cytoplasm [28]. Different PHDs have different roles. PHD2 is a key regulator of HIF-α expression [29], while the relative abundance of PHD3 and PHD1 may determine the selectivity of HIF-1α and HIF-2α expression [30]. What’s more, PHD2 seems to be the hydroxylase that is essential for HIF-α degradation during normoxia [29,31], whereas PHD3 appears to play an important role in hydroxylating HIF-α under reoxygenation [30].

In addition to PHDs, factor inhibiting HIF (FIH) is also a vital oxygen-sensitive enzyme for HIF regulation. With oxygen, FIH hydroxylates an HIF-α’s asparaginyl residue to prevent the recruitment of the CREB-binding protein (CBP)/p300 coactivators, which is required for the full transcriptional activity of HIF [32,33]. Thus, PHDs control the stability or expression level of HIFαs, while FIH1 regulates HIF’s transcription activity, providing a dual mechanism of HIF upregulation in response to hypoxia (Figure 1). Of note, FIH1 remains active at lower oxygen concentrations than PHDs, and may therefore inhibit the activity of HIF that has escaped PHD-mediated destruction during moderate hypoxia [34,35]. Under hypoxia, HIF-α becomes stable and translocates into the nucleus, and then dimerizes with HIF-β to transactivate target genes. Meanwhile, FIH inactivation promotes CBP/p300 recruitment for increasing the transcriptional activity of HIF [31]. In the kidneys, FIH has been detected in the distal tubules and podocytes [36].

Besides the classic regulation by PHDs and FIH, the stability, nuclear accumulation, and transcriptional activity of HIF-α are also modulated by other signaling pathways. Recent studies have indicated that direct phosphorylation of HIF-α plays an important role in regulating HIF-α stability, nuclear localization, as well as transcription activity [37]. Both glycogen synthase kinase-3beta (GSK-3β) and polo-like kinase 3 (Plk3) can enhance HIF-α degradation by directly phosphorylating the HIF-α proteins [38,39]. In contrast, protein kinase A (PKA) may phosphorylate HIF-1α to inhibit its proteasomal degradation and enhance its transcriptional activity [40]. Mylonis et al. demonstrated that phosphorylation of HIF-1α by MAPK/ERK promoted its nuclear accumulation and transcriptional activity through blocking CRM1-dependent nuclear export [41,42]. Interestingly, ERK-dependent activation of HIF-1 could be inhibited by HIF-1α-derived cell-penetrating peptides [43]. Kalousi et al. found that casein kinase 1delta (CK1δ) phosphorylated HIF-1α to prevent its association with HIF-β and attenuate HIF-1 activity [44]. CK1δ could also phosphorylate HIF-2α; however, this phosphorylation promoted the nuclear accumulation and transcription activity of HIF-2α [45].

A growing number of studies, even if being conflicting, have indicated that reactive oxygen species (ROS) can regulate HIF-α under normoxia and hypoxia [46,47,48]. Under normoxia, ROS appear to act as signaling molecules for HIF-1α [49]. Hypoxia results in increased production of ROS at the electron transport chain, which may increase HIF-1α’s stability and activity by inhibiting PHD’s activity [50,51]. Additional studies showed that increased ROS production could prevent hydroxylation and degradation of HIF-1α and HIF-2α [52]. However, other studies demonstrated that increased ROS promoted the degradation of HIF-1α via the ubiquitin–proteasome system [48]. Thus, the regulation of HIF by ROS may depend on cellular and experimental context [47,52,53,54]. Moreover, it may involve the feedback through nuclear factor kappa B (NF-κB) and Nrf2 [51,55,56].

In addition, heat shock protein 90 (HSP90) inhibitors and histone deacetylase inhibitors (HDACIs) were reported to accelerate HIF-α degradation in a manner independent of pVHL [57,58]. Receptor of activated protein kinase C 1 (RACK1) may prompt HIF-1α degradation via competing with HSP90 [59]. Under certain conditions, nitric oxide (NO)-mediated S-nitrosylation can enhance the stability and activity of HIF-1α [60]. In addition, sumoylation also plays a role in regulating HIF-α stability [61] (Figure 1).

## 3. Expression Patterns and Functions of HIFs

There are three subtypes of HIFs named HIF-1, HIF-2, and HIF-3 due to the labile subunit HIF-α (HIF-1α, HIF-2α, or HIF-3α). HIF-1α is ubiquitously expressed in organs of most cell types, whereas HIF-2α expression is tissue limited and detected particularly in highly vascularized tissues and organs [62,63]. In kidney, HIF-1α is found in most renal epithelial cells, while HIF-2α is mainly expressed in renal interstitial fibroblast-like cells and endothelial cells. In addition, HIF-1α is also detected in endothelial and interstitial cells of the papilla and inner medulla, but not in the outer medulla and cortex [64].

In general, both HIF-1α and HIF-2α are activated by hypoxia but in different phases: HIF-1α takes part in the initial adaptation process of hypoxia as it is rapidly induced and then falls to a low level within 72 h, whereas HIF-2α accumulation begins under prolonged hypoxic conditions [65,66,67]. Upon prolonged hypoxia, upregulated natural antisense RNA of HIF-1α may destabilize HIF-1α’s mRNA to decrease its expression in lung epithelial cells, whereas the expression of *HIF-2α* is not affected [68]. Chen et al. showed that the elevated HIF-1α under chronic hypoxic pulmonary hypertension may activate the transcription of *PHD2* and *PHD3*, suggesting that upregulated *PHDs* under chronic hypoxia may act as a negative feedback mechanism for *HIF-1α* [69]. Later research indicates that *LIMD1*, a HIF-1 target gene, mediates a negative feedback for HIF-1α degradation under chronic hypoxic conditions by modulating PHD2–LIMD1–VHL complex formation [70].

Functionally, HIF-1 plays an important role in regulating metabolism. It not only mediates the transition from oxidative metabolism to glycolysis to generate ATP in an oxygen-independent manner, but also mediates the subunit conversion in cytochrome c oxidase under hypoxic conditions to improve the efficiency of electron transfer [71,72]. In addition, HIF-1 also takes part in the regulation of fibrosis, cell death, and inflammation [73], although the underlying mechanisms are less clear. HIF-2 is a major regulator of erythropoietin production [16] and vessel remodeling in diseases [74,75]. In contrast, the function of HIF-3 remains largely unknown and controversial. There are studies that suggest full-length HIF-3 may act as an oxygen-regulated transcriptional activator for a unique transcriptional program in response to hypoxia. On the other hand, some short HIF-3 variants may suppress *HIF-1* and *HIF-2* by acting as dominant-negative inhibitors that compete for *HIF-β* [76]. In addition, HIF-1 can transcriptionally activate the expression of *HIF-3α*, adding another layer of complexity [77].

## 4. Well-Known HIF Target Genes and Their Functions in Kidney

Under hypoxia, HIFs accumulate and bind to the hypoxia response elements (HRE) in the enhancer or promoter region of their target genes, resulting in transcription [20,26,78]. Both HIF-1 and HIF-2 promote oxygen delivery and cellular adaptation to hypoxia via stimulating multiple cellular and tissue responses, including erythropoiesis, angiogenesis, anaerobic glucose metabolism, iron metabolism, and adenosine and NO metabolism [18,19]. Among them, erythropoiesis, angiogenesis, and anaerobic glucose metabolism, which are respectively regulated by *EPO*, *VEGF*, and glycolytic genes, are the most important hypoxia responses in kidney injury and repair. There is evidence that glycolytic genes are predominantly regulated by HIF-1 [79], whereas *VEGF* and *EPO* induction are preferentially regulated by HIF-2 [80,81,82]. Interestingly, in cells lacking HIF-1, there is no induction of hypoxia responsive genes, suggesting that HIF-1 is a prerequisite for inducing this family of genes in some cells [83].

### 4.1. Erythropoietin (EPO)

EPO, a hematopoietic growth factor secreted by the kidney and liver, promotes red blood cells generation (erythropoiesis) in the bone marrow, thus enhancing the blood’s oxygen carrying capacity [72]. Upon hypoxia, HIF accumulates and binds to the HRE of *EPO* in the 3′ enhancer region [20,84]. The chief function of EPO is to promote erythropoiesis. In the regulation of erythropoiesis, kidney is the most important oxygen sensor, which responds to systemic hypoxia, and then increase the production of EPO rapidly by renal interstitial fibroblast-like cells [85,86]. Liver can also produce EPO to promote erythropoiesis in an oxygen-dependent mode, but it is not sufficient to compensate the loss of kidney EPO in end-stage renal disease, leading to anemia that requires systemic treatment with recombinant EPO [87]. In addition, EPO can also protect against kidney injury by reducing apoptosis and inflammation, and increasing tubular cell proliferation [88].

### 4.2. Vascular Endothelial Growth Factor (VEGF)

VEGF, induced by hypoxia or ischemia, plays an important role in angiogenesis by activating the receptor tyrosine kinases (*VEGFR-1*, *VEGFR-2*, and *VEGFR-3*) [89,90]. The function of renal glomerulus is dependent on the specialized vasculature maintained by VEGF [91]. Overexpression of podocyte-derived *VEGF* in glomeruli leads to a collapsing glomerulopathy [92], whereas suppression of podocyte *VEGF* expression destroys the filtration barrier, resulting in protein leakage and glomerular thrombotic microangiopathy (TMA) [93].

## 5. HIF in AKI and Mechanisms of HIF Signaling in AKI

Depending on the condition of perfusion, the oxygen supply to the kidneys, especially the cortex, can vary significantly. Notably, the renal proximal tubule cells have very limited capacity of ATP production via anaerobic glycolysis, resulting in rapid consumption of, and high dependence on, oxygen in maintaining oxidative metabolism. These make the kidney susceptible to hypoxic damage. In hypoxia (or ischemia in vivo), HIFs play an important role in the pathogenesis of AKI.

### 5.1. HIF in IR-Induced AKI

Renal ischemia-reperfusion injury (IRI) is one of the main causes of AKI associated with a variety of clinical conditions, such as kidney transplantation, renal vascular occlusion, and cardiac arrest resuscitation [94]. The involvement of HIFs in kidney IRI has been demonstrated in numerous studies. Both ischemic pre-conditioning (caused by short-term ischemia) and hypoxia pre-conditioning (caused by carbon monoxide, which reduces tissue oxygen availability through blocking the oxygen carrying capacity of hemoglobin) can induce HIF, leading to resistance against subsequent IR injury [95,96]. Activating *HIF-1α* and *HIF-2α* by pretreatment with pharmacological PHDs inhibitors significantly reduced ischemic kidney injury by reducing apoptosis, macrophage infiltration, and vascular cell adhesion molecule 1 (*VCAM1*) expression, and upregulating HIF target genes [21,96,97,98,99,100,101]. However, the effect of post-ischemic treatment with pharmacological PHDs inhibitors is controversial. Jamadarkhana et al. found that administrating PHD inhibitor TRC160334 at 2 h, 6 h, and 10 h post the onset of kidney ischemia activated the expression of *HIF-1* and attenuated kidney injury by inducing heat shock protein 70 (HSP70) [102]. Also, administrating granulocyte colony-stimulating factor (G-CSF) and stem cell factor (SCF) 6 h after IRI also activated the expression of *HIF-1* and reduced the degree of kidney tissue injury by upregulating the expression of *VEGF* and *EPO* [103]. But, other studies demonstrated that administrating PHD inhibitors after renal ischemia had no effects in attenuating AKI and renal fibrosis [99,100]. There are several possible causes of the apparent discrepancy between these studies [99,100,102]: (1) the frequency of the administration of PHD inhibitors—the research by Jamadarkhana et al. [102] involved repetitive application of PHD inhibitor, while the research by Wang et al. [99] included only single application; (2) the method of the administration of PHD inhibitors—the PHD inhibitor was administered by oral gavage by Kapitsinou et al. [100], while the PHD inhibitor was injected by Jamadarkhana et al. [102]; (3) Jamadarkhana et al. [102] tested various doses, whereas Wang et al. and Kapitsinou et al. [99,100] tested only a single dose; and (4) the time of the administration of PHD inhibitors. Thus, there may be a narrow therapeutic window of PHD inhibitors for treatment when given post ischemia.

Conde et al. showed that short interfering RNA (siRNA) against HIF-1α exacerbated renal IR injury [11]. A later study further demonstrated that inhibiting *HIF-1α* by siRNA during reperfusion had deleterious effects on kidney injury and renal fibrosis by downregulating *miR-127-3p* and inducing its target gene *Bcl6* [104]. Meanwhile, Zhang et al. discovered that *HIF-1α* siRNA counteracted the protective effect of isoflurane on renal ischemia-reperfusion injury [105]. Hill et al. found that heterozygous *HIF-1α* and *HIF-2α* knockdown (KO) mice had more pronounced renal IR injury than wild-type littermates [98]. Using *HIF-2α* knockdown mice, Kojima et al. demonstrated that HIF-2 protected against renal ischemia by ameliorating oxidative stress [106]. Remarkably, Kapitsinou et al. analyzed the functions of *HIF-1* and *HIF-2* in endothelial cells during renal IR injury by testing conditional endothelial cell (EC)-specific *HIF-1α* and *HIF-2α* double or single knockout mouse models, demonstrating that endothelial HIF-2, but not endothelial HIF-1, protected from renal ischemia-reperfusion injury by reducing the expression of *VCAM1* [21].

Mechanistically, apart from the aforementioned mechanisms such as oxidative stress, upregulating HIF target genes, and reducing *VCAM1* expression, several studies have demonstrated that HIF-induced microRNAs play important roles in ischemic AKI. MicroRNAs are short non-coding RNAs that control post-transcriptional gene expression by translational inhibition and/or mRNA degradation through binding to target gene mRNA. For example, our recent study showed that microRNA-489 is induced via HIF-1 in ischemic AKI to protect renal proximal tubules by targeting relevant genes [107]. Our latest research further demonstrated that HIF-1 induces *miR-668* to protect against ischemic AKI via repressing *MTP18* to preserve mitochondrial dynamics [108]. Conde et al. showed that downregulating *miR-127-3p* by HIF-1α interference may promote induction of collagen I and α-SMA, and loss of E-cadherin [104]. Meanwhile, HIF-1 may also protect against IR-induced kidney injury via *miR-21* target pathways [101,109], further indicating that HIF may protect kidneys via microRNAs. On the other hand, HIF can also induce the expression of injurious microRNAs. For example, *miR-687* is induced via HIF-1 in proximal tubule cells in ischemic AKI, and blocking *miR-687* attenuated kidney injury by preserving *PTEN* expression and attenuating renal apoptosis and cell cycle activation [110]. Thus, in addition to the induction of protective microRNAs, HIF may also induce injurious microRNAs. Regardless, all these studies supported an important role of HIFs in the pathogenesis of ischemia AKI. Interestingly, a recent study by Mathia and colleagues showed that *miR-22* was induced to repress *HIF-1α* in a mouse model of rhabdomyolysis-associated AKI. Specific antagonism of *miR-22* resulted in *HIF-1α* upregulation, but it was unable to ameliorate AKI likely due to the expression of both protective and injurious genes [111].

### 5.2. HIF in Cisplatin-Induced AKI

Induction and activation of *HIF* have also been implicated in the pathogenesis of nephrotoxic AKI. One such example is AKI induced by cisplatin, a chemotherapeutic drug widely used to treat various malignancies. During cisplatin treatment of cancer, more than one-quarter of patients developed kidney problems, especially AKI [112]. In addition to causing renal tubule cell death, cisplatin also induced vascular dysfunction, resulting in hypoperfusion or hypoxia in kidneys [113]. In vitro, proximal tubular cells preconditioned with hypoxia reduced cisplatin-induced apoptosis in HIF-1α-dependent manner. In addition, in vivo, rats preconditioned with carbon monoxide prior to cisplatin administration ameliorated cisplatin-induced AKI [114]. Consistently, activating *HIF-1* by pretreatment with pharmacological PHDs inhibitors such as cobalt and FG-4592 significantly attenuated cisplatin-induced AKI by inhibiting mitochondrial signaling pathways and upregulating HIF target genes [22,115]. In addition to drug intervention, implantation of stem cells had sparked great interest in the area of kidney repair following AKI. Wang and colleagues found that human adipose-derived stem cells (hASCs) transfected with *HIF-1α* provided obvious protective effects against cisplatin-induced kidney injury on tubular structure and renal function by suppressing inflammation, reducing renal tubular apoptosis and upregulating heme oxygenase 1 (*HO-1*) gene expression [116]. What’s more, delayed administration of lithium promoted recovery from cisplatin or IRI-induced AKI by stabilizing pro-proliferative molecules including HIF-1α [117].

In conclusion, these findings indicated that intrinsic HIF activation, even at a modest level, may benefit kidneys during cisplatin chemotherapy.

### 5.3. HIF in Sepsis-Associated AKI

Sepsis is a systemic inflammatory response caused by infection and is the most common contributing factor in the development of AKI [118]. The systemic inflammatory response can be initiated by bacterial lipopolysaccharide (LPS) and/or other microbial component into the lymph and circulatory system. Early research indicated that pre-conditioning with chronic hypoxia protected against LPS-induced AKI by attenuating oxidative stress and inflammatory cytokine release via enhancing the ratio of intrarenal antioxidant/oxidative protein [119]. In septic patients, EPO and HIF-1α play important roles in the pathogenesis of sepsis-AKI [120]. He and colleagues reported that pre-conditioning with LPS led to HIF-2α accumulation via NF-κB in endothelial cells, which was responsible for the resistance of the pre-conditioned mice to subsequent ischemic AKI [121]. Importantly, Stoyanoff et al. indicated that phosphorylated *NF-κB*, *p65*, and *HIF-1α* were simultaneously overexpressed in LPS-induced renal damage, and EPO administration attenuated septic-AKI through decreasing *HIF-1α* and *NF-κB* expression [122]. Consistently, landiolol hydrochloride, acting as an ultra-short-acting beta-blocker, attenuated LPS-induced AKI by ameliorating *HIF-1* upregulation and normalizing inflammatory cytokines such as *TNF-α* [123]. Together, these results suggested that the activation of *HIF-1* may be harmful during septic or LPS-induced AKI.

### 5.4. HIF in AKI Induced by Other Causes

There are reports that HIF also takes part in AKI induced by rhabdomyolysis [23], gentamicin [124], and contrast medium in combination of nitric oxide synthase (NOS) inhibitor and cyclooxygenase inhibitor [125]. Selectively activating *HIF* in renal tubules by Pax8-rtTA-based inducible knockout of von Hippel–Lindau protein (VHL-KO) protected against rhabdomyolysis-induced renal damage through a metabolic sHIFt to anaerobic energy metabolism [23]. In gentamicin-induced AKI, activating *HIF-1* by continuous infusion of cobalt ameliorated renal damage by reducing renal tubular apoptosis and macrophage infiltration [124]. Early studies indicated that contrast medium could reduce renal oxygen tension via increasing tubular oxygen consumption for solute reabsorption and decreasing renal perfusion by vasoconstriction and peritubular capillaries compression [126,127]. In a multi-insult-AKI rat model caused by contrast medium, NOS inhibitor, and COX inhibitor, furosemide treatment ameliorated kidney injury with an unexpected HIF-1α increase [125]. Thus, *HIF* induction is a common observation in various AKI models. Following induction, HIF generally has a renoprotective role but it may contribute to AKI under specific conditions (Table 1).

## 6. Role of HIF in Kidney Repair

After injury, the kidney has the capacity of repair. This is particularly true for renal tubules. If the initial injury is mild, kidney repair can be complete resulting in intact, fully functional repair; however, if the initial injury is severe, kidney repair is incomplete and may lead to chronic pathologies and gradual decline of renal function. HIFs have been implicated in kidney repair in various models (Table 2).

### 6.1. Integral Introduction of Kidney Repair

After AKI, the kidneys have the capability to repair damaged tubules. Depending on the severity of injury, the repair can be complete or incomplete. Complete repair (also called “adaptive repair”) can restore the integrity and function of renal tubules, whereas incomplete or maladaptive repair, characterized by undifferentiated and atrophic tubules and persistent inflammation, leads to renal interstitial fibrosis and possible progression to CKD [6,7,128,129]. Tubulointerstitial hypoxia is considered to be a common pathway for progressive kidney disease. Hypoxia inhibits the growth of renal tubular epithelial cells and results in failure of remodeling by accelerating dedifferentiation and apoptosis [130,131]. In addition, hypoxia can also convert tubular epithelial cells to a pro-fibrotic phenotype [132] and promote tubulointerstitial inflammation [133]. Human microarray data have identified the hypoxia target genes in cell death (*GsE12546*), cellular proliferation (*GsE4725*), differentiation (*GsE9510* and *GsE4630*) and inflammation (*GsE968* and *GsE10723*) [134]. Although resident renal cells upregulate *HIFs* and relevant genes upon hypoxia, hypoxia adaptation through *HIF* is not sufficient in CKD as *HIF* is suppressed by many factors such as uremia and oxidative stress [135]. A list of in vivo studies on the effect of HIFs in kidney repair is presented in Table 2.

### 6.2. HIF in Kidney Cell Death, Dedifferentiation, and Proliferation

One mechanism for HIF involvement in kidney repair is by regulating kidney cell death. It is well known that appropriate kidney repair involves a precise balance between renal tubular cell death and proliferation [136]. Hypoxia may induce cell death of renal tubules and, to a limited extent, endothelial cells [131,137]. HIF-1α affects cell death by regulating Bcl-2 family genes, interacting with p53, and/or targeting mitochondria enzymes [138]. A large amount of work has demonstrated that *HIF* activation decreases renal apoptosis in different kinds of AKI models [96,98,114,115,116]. Consistently, inhibiting *HIF-1* in vitro by HIF-1α interference promoted cell death [11]. In chronic models of progressive Thy1 nephritis and the remnant kidney, activating *HIF* by cobalt also reduced apoptotic tubular cells [139,140]. In addition, upregulated *HIF-1α* promoted kidney tissue repair from acute tubular necrosis [103].

In non-lethal injury, proliferation and re-differentiation of proximal tubule cells are the major contributors to tubular repair [141]. Maladaptive tubular cells’ repair may occur when epithelial cells are not fully re-differentiated [142] or arrested in G2 phase of cell cycle [143]. Many groups have indicated that surviving tubular cells could transiently upregulate tubular epithelial cell dedifferentiation markers such as alpha-smooth muscle actin, vimentin, and S100A4. Co-expression of PCNA-proliferating cell nuclear antigen with these markers indicated that the dedifferentiated cells are actively replicating [144,145]. In addition, The dedifferentiated cells respond to diverse proliferative signals, including epidermal growth factor (EGF), hepatocyte growth factor (HGF), transforming growth factor β (TGF-β), and VEGF [146]. Under this condition, HIF-1α may facilitate cell proliferation [147]. *HIF-1α* induction during reperfusion acts as a key factor in proximal epithelial cell regeneration by promoting the expression of tissue repair genes [11]. In the remnant kidney model, *HIF* activation preserved the peritubular capillary networks by increasing the number of proliferating glomerular and peritubular endothelial cells [140]. Mechanistically, HIF-1 can enhance tissue repair by upregulating *EPO* to stimulate cellular regeneration as well as inhibit apoptotic cell death [148] and induce stromal cell-derived factor-1 (*SDF-1*) to promote recruitment of progenitor cells for regenerating tissues [149]. On the other hand, HIF-1α can also inhibit cell proliferation [150]. A recent study reported that the induction of *HIF-1α* during hypoxia inhibited the proliferation of mesenchymal stem cells through increasing the cell cycle inhibitor p27 [151]. What’s more, *HIF-1α* inhibition by siRNA induced proximal tubule cells proliferation during renal I/R [104]. Thus, in terms of tubular cell proliferation, HIF may be a double-sided sword.

### 6.3. HIF in Kidney Fibrosis

Maladaptive repair post-AKI leads to renal interstitial fibrosis [6]. Increasing evidence indicates that HIF is a pivotal regulator of kidney fibrosis under various pathological conditions [21,100,152]. However, it remains controversial whether HIF promotes or antagonizes renal fibrosis. In 2007, Higgins et al. demonstrated a critical role of renal tubular HIF-1 in renal fibrosis during unilateral ureteral obstruction (UUO) [24]. In ischemia-reperfusion injury, Kapitsinou et al. showed that activating *HIF* by pharmacological PHD inhibitor GSK1002083A before ischemia ameliorated AKI-induced fibrosis, but post-ischemic *PHD* inhibition had no effect on renal fibrosis [100]. In remnant kidney model, pharmacological activating *HIF* ameliorated tubulointerstitial injury and decreased fibrosis [140,153]. Further research indicated that the renoprotective effect of HIF in the remnant kidney model relied on the timing and isoform of *HIF* activation [154]. Administrating a PHD inhibitor (HIF stabilizer) at an early stage accelerated renal fibrosis, while administrating at a more advanced stage decreased renal fibrosis. Notably, the PHD inhibitor given at early stage activated both *HIF-1α* and *HIF-2α*, whereas PHD inhibitor given later only activated *HIF-2α* with no effect on *HIF-1α* [154]. The time-dependent effects suggest that renal fibrosis in this model may be related to the activation of specific *HIF* isoforms in specific cells. In the model of hypertensive type 2 diabetes, pharmacological activating *HIF* by CoCl_2_ also attenuated renal fibrosis [155]. In contrast, Conde et al. found that *HIF-1α* siRNA increased the expression of fibrotic markers and promoted the epithelial-to-mesenchymal transition (EMT) process in renal I/R [104]. Furthermore, Kobayashi and colleagues indicated that genetic activation of *HIF* suppressed fibrogenesis in UUO [156]. In addition, using genetic methods, Kapitsinou et al. demonstrated that inhibiting endothelial *HIF* accelerated renal fibrosis both in IR model and UUO model [21].

On the contrary, there are many studies demonstrated that HIF was pro-fibrotic in kidney. Kimura and his colleagues found that injection of a pharmacologic HIF-1 inhibitor (3-(5’-hydroxymethyl-2’-furyl)-1-benzyl indazole) decreased renal fibrosis in UUO [157]. In rat angiotensin II-induced renal injury and chronic ischemic renal injury, the increase of fibrotic proteins (α-smooth muscle actin and collagen) was blocked by *HIF-1α* shRNA [152,158]. Consistently, in hypoxia/reoxygenation model, persistent activation of *HIF-1α* by using an HIF-1αΔODD-expressing adenovirus significantly increased the expression of α-smooth muscle actin and decreased the expression of E-cadherin [159]. Genetic ablation of proximal tubule epithelial *HIF-1α* impeded the development of kidney fibrosis in UUO model [24]. Similarly, genetic overexpression of *HIF-1α* in tubular epithelial cells by von Hippel–Lindau tumor suppressor (*VHL*) deletion exacerbated interstitial fibrosis in a 5/6 renal ablation model [157]. Mechanistically, HIF signaling may promote renal fibrosis via at least four mechanisms: (1) transcriptional regulation of fibrogenic genes; (2) cross-talk with other pro-fibrotic signaling pathways such as TGF-β, NF-κB, Notch, and PI3K/Akt pathways; (3) its potential role in EMT; and (4) epigenetic regulation [73].

Collectively, these studies demonstrate an important regulatory role of HIF in renal fibrogenesis. However, whether HIF is pro- or anti-fibrotic may depend on which, where, and when HIF is activated.

### 6.4. HIF in Kidney Inflammation

**Figure 2:**
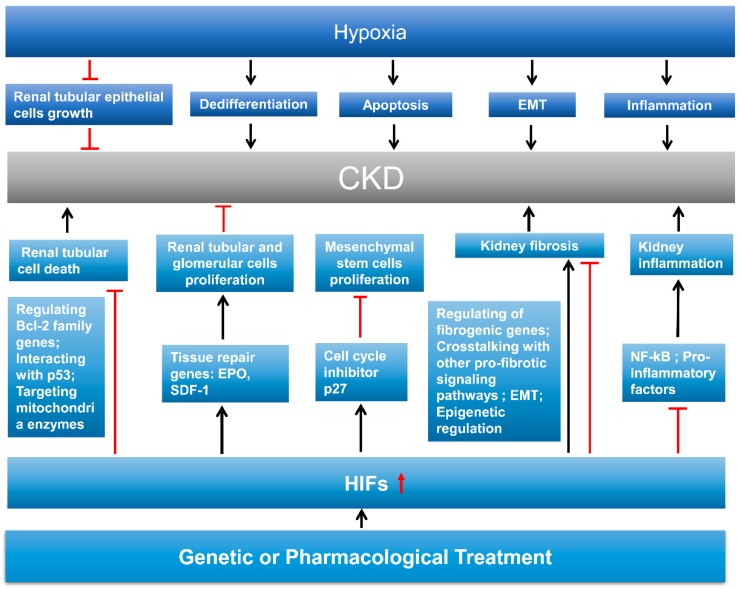
Role of hypoxia and HIF in kidney repair. Hypoxia accelerates the progression of CKD by inhibiting renal tubular epithelial cell growth and promoting dedifferentiation, apoptosis, EMT, and inflammation. Upregulation of HIFs by genetic or pharmacological treatment may (1) inhibit renal tubular cell death by regulating Bcl-2 family genes, interacting with p53, and/or targeting mitochondria enzymes; (2) promote renal tubular and glomerular cell proliferation by inducing tissue repair genes such as *EPO* and *SDF-1*; (3) inhibit mesenchymal stem cell proliferation by increasing the cell cycle inhibitor *p27* expression; (4) promote or inhibit kidney fibrosis by regulating fibrogenic genes, cross-talking with other pro-fibrotic signaling pathways, EMT, and epigenetic regulation; and (5) inhibit kidney inflammation by reducing the expression of *NF-κB* and pro-inflammatory factors. Abbreviations: epithelial-to-mesenchymal transition (EMT), chronic kidney disease (CKD), erythropoietin (EPO), stromal cell-derived factor-1 (SDF-1), and hypoxia-inducible factor (HIF).

Persistent inflammation is a characteristic of maladaptive kidney repair post AKI. Interestingly, inflammation and hypoxia often coexist and have been shown to regulate each other. On one hand, hypoxia and HIF-1 strongly influence inflammatory cell recruitment [160] and function [161]. On the other hand, inflammatory cells regulate the activation of hypoxic signaling pathways [162]. In the unilateral ischemia-reperfusion (uIR) and remnant kidney models, HIF activation by cobalt chloride decreased macrophage infiltration [97,140]. A latter study indicated that macrophages exhibited phenotype sHIFt (from M1 to M2) during the kidney repair process [163]. In a rat renal IR model, silencing *HIF-1α* exacerbated inflammatory response by activating *NF-κB* and inducing pro-inflammatory factors [104]. In a mouse model of UUO, global or conditional knockout of *HIF-1* and *HIF-2* in myeloid cells caused more severe inflammation. In the meanwhile, activating *HIF* via myeloid-specific VHL-knockout suppresses inflammation [156]. Moreover, conditional knockout of endothelial *HIF-2α*, rather than *HIF-1α*, led to enhanced inflammatory cell infiltration in both uIR and UUO mouse models [21]. On the contrary, in renal epithelial cells, genetic ablation of *HIF-1α* decreased inflammatory cell infiltration in mice UUO model [24]. Together, these studies indicate that HIF may play a modulatory role in inflammation in kidney injury and repair. HIF may suppress inflammation directly by working in immune cells and indirectly by working in epithelial and endothelial cells (Figure 2).

## 7. Therapeutic Potential of HIF in AKI and CKD

HIF is the master switch for hypoxic adaptation in cells and tissues. In kidneys, as in other organs, HIF increases oxygen supply and improves the tolerance to conditions of hypoxia or ischemia in vivo. Hypoxia occurs frequently in diseased kidneys and HIF is often suboptimal under these conditions. This is particularly true for AKI and post-AKI kidneys under repair. Thus, there is a great therapeutic potential by targeting or activating HIF. As discussed above, HIF is primarily modulated by the PHD–VHL pathway. A major breakthrough in clinical application is that multiple clinical trials have shown the therapeutic effects of PHD inhibitors for anemia in CKD patients. The first PHD inhibitor, roxadustat, was recently approved for clinical use in China. Of note, these are Pan-PHD inhibitors and do not have specificity for a specific PHD isoform. There are three PHD isoforms that have different characteristics, including target selectivity for *HIF-α* isoforms and expression levels in cell types. Therefore, there is an urgent need to develop agents that can specifically inhibit each *PHD* isoform that are expected to be safer and have specific therapeutic spectrum. In addition, excessive activation of *HIF* may sometimes have deleterious effects. It is therefore important to systematically titrate and optimize the degree, timing, and duration of *HIF* activation. Finally, despite various experimental studies, it remains unclear if targeting HIF-PHD is an effective treatment for kidney diseases in human patients. In this regard, we expect serious efforts in both experimental and clinical test of PHD inhibitors in coming years.

## Figures and Tables

**Table 1:** Summary of in vivo studies on the effect of HIFs in kidney injury.

AKI Model	Approach for HIF Activation/Inhibition	Which HIF Was Activated/Inhibited	Effects on Kidney Injury	Mechanisms	References
IRI in mice	15 min renal ischemic pre-conditioning	*HIF-1* was activated	Attenuate AKI	Increasing the expression of *miR-21*	[95]
uIRI in rat	Carbon monoxide	*HIF-1* and *HIF-2* were activated	Attenuate AKI	Alleviating apoptosis and macrophage infiltration	[96]
uIRI in mice	PHD inhibitor	*HIF-1* and *HIF-2* were activated	Attenuate AKI	Alleviating apoptosis and macrophage infiltration	[98]
IRI in rat	PHD inhibitor	*HIF-1* and *HIF-2* were activated	Attenuate AKI	Upregulating HIF target genes, including *EPO*	[99]
uIRI in mice	PHD inhibitor	*HIF-1* and *HIF-2* were activated	Attenuate AKI	Reducing *VCAM1*	[21]
IRI in mice	PHD inhibitor	*HIF-1* and *HIF-2* were activated	Attenuate AKI and renal fibrosis	Reducing inflammation	[100]
uIRI in rat	Cobalt chloride	*HIF-1* was activated	Attenuate AKI	Inducing renoprotective gene expression	[97]
IRI in mice	Cobalt chloride	*HIF-1* was activated	Attenuate AKI	Upregulating *VEGF* and *miR-21*	[101]
IRI in rat	HIF-1α siRNA	*HIF-1* was inhibited	Aggravate AKI		[11]
IRI in mice	*HIF-1α*(+/−) or *HIF-2α*(+/−) mice	*HIF-1* or *HIF-2* was inhibited	Aggravate AKI		[98]
IRI in mice	*HIF-2α* knockdown mice	*HIF-2* was inhibited	Aggravate AKI	Enhancing oxidative stress	[106]
uIRI in mice	EC-specific *PHD2*−/− mice	Endothelial *HIF* was activated	Attenuate kidney injury	Reducing *VCAM1*	[21]
uIRI in mice	EC-specific *HIF2α*−/− mice with PHD inhibitor	*HIF-1* was activated	Ineffective in attenuating AKI		[21]
Cisplatin-AKI in rat	Cobalt	*HIF-1* was activated	Attenuate AKI	Inhibiting mitochondrial signaling pathways	[115]
Cisplatin-AKI in mice	PHD inhibitor	*HIF-1* was activated	Attenuate AKI	Upregulating HIF target genes	[22]
LPS-AKI in rat	Landiolol hydrochloride	Ameliorate the upregulation of *HIF-1*	Attenuate AKI	Normalizing inflammatory cytokines	[123]
Rhabdomyolysis-AKI in mice	Pax8-rtTA–based inducible VHL-KO	Renal tubules HIF was activated	Attenuate AKI	Metabolic sHIFt toward anaerobic energy metabolism	[23]
Gentamicin-AKI in rat	Cobalt	*HIF-1* was activated	Attenuate AKI	Reducing apoptosis and macrophage infiltration	[124]
Multi-insult-AKI in rat(contrast medium, NOS inhibitor, and COX inhibitor)	Furosemide	*HIF-1* was activated	Attenuate AKI	Upregulating *HO-1*	[125]

AKI, acute kidney injury; HIF, hypoxia-inducible factor; IRI, ischemia-reperfusion injury; uIRI, unilateral ischemia-reperfusion injury; PHD, prolyl hydroxylase domain-containing protein; EPO, erythropoietin; VCAM1, vascular cell adhesion molecule-1; VEGF, vascular endothelial growth factor; siRNA, short interfering RNA; EC, endothelial cell; LPS, lipopolysaccharide; VHL-KO, knockout of von Hippel–Lindau protein; NOS, nitric oxide synthase; COX, cyclooxygenase; HO-1, heme oxygenase 1.

**Table 2:** Summary of in vivo studies on the effect of HIFs in kidney repair.

AKI Model	Approach for HIF Activation/Inhibition	Which HIF was Activated/Inhibited	Effects on Kidney Repair	Mechanisms	References
IRI in rat	PHD inhibitor	*HIF-1* was activated	Attenuate AKI	Inducing HSP70	[102]
IRI in rat	SCF and G-CSF	*HIF-1* was activated	Attenuate AKI	Upregulating *VEGF* and *EPO*	[103]
IRI in rat	*HIF-1α* siRNA	*HIF-1* was inhibited	Aggravate AKI and renal fibrosis	Downregulating *miR-127-3p* and inducing its target gene *Bcl6*	[104]
IRI in rat	PHD inhibitor	*HIF-1* and *HIF-2* were activated	Ineffective in attenuating AKI		[99]
IRI in mice	PHD inhibitor	*HIF-1* and *HIF-2* were activated	Ineffective in attenuating AKI and renal fibrosis		[100]
IRI in rat	HIF-1α siRNA	*HIF-1* was inhibited	Aggravate AKI		[11]
uIRI in mice	EC-specific *HIF-1a HIF-2α*−/− mice	Endothelial *HIF-1* and *HIF-2* were inhibited	Impair kidney recovery and worsen renal fibrosis	Activating *VCAM1*	[21]
uIRI in mice	EC-specific HIF-1α−/− or HIF2α−/− mice	Endothelial *HIF-1* or *HIF-2* was inhibited	Inactivation of endothelial *HIF-2* but not *HIF-1* impairs kidney recovery	Activating *VCAM1*	[21]
Cisplatin-AKI in mice	Lentivirus-mediated *HIF-1α*-transfected hASCs	*HIF-1* was activated	Attenuate AKI	Upregulating *HO-1*	[116]
LPS-AKI in mice	*EPO*	*HIF-1* was inhibited	Attenuate AKI	Promoting angiogenesis	[122]

AKI, acute kidney injury; HIF, hypoxia-inducible factor; IRI, ischemia-reperfusion injury; PHD, prolyl hydroxylase domain-containing protein; HSP70, heat shock protein 70; SCF, stem cell factor; G-CSF, granulocyte colony-stimulating factor; VEGF, vascular endothelial growth factor; EPO, erythropoietin; siRNA, short interfering RNA; uIRI, unilateral ischemia-reperfusion injury; EC, endothelial cell; VCAM1, vascular cell adhesion molecule-1; hASCs, human adipose-derived stem cells; HO-1, heme oxygenase 1; LPS, lipopolysaccharide.
